# Agricultural input credit in Sub-Saharan Africa: Telling myth from facts

**DOI:** 10.1016/j.foodpol.2016.09.014

**Published:** 2017-02

**Authors:** Serge G. Adjognon, Lenis Saweda O. Liverpool-Tasie, Thomas A. Reardon

**Affiliations:** a World Bank Group (WBG), Development Impact Evaluation Unit (DIME), Development Research Group (DECRG), USA; b Department of Agricultural, Food, and Resource Economics, Michigan State University, East Lansing, MI, USA

**Keywords:** Africa, Farm inputs, Credit, Rural nonfarm employment

## Abstract

Recent evidence shows that many Sub-Saharan African farmers use modern inputs, but there is limited information on how these inputs are financed. We use recent nationally representative data from four countries to explore input financing and the role of credit therein. A number of our results contradict “conventional wisdom” found in the literature. Our results consistently show that traditional credit use, formal or informal, is extremely low (across credit type, country, crop and farm size categories). Instead, farmers primarily finance modern input purchases with cash from nonfarm activities and crop sales. Tied output-labor arrangements (which have received little empirical treatment in the literature) appear to be the only form of credit relatively widely used for farming.

## 1 Introduction

It is generally accepted that Sub-Saharan Africa (SSA) farmers often have low yields which could be increased, all else equal if they bought more “external inputs” (chemical fertilizer, pesticides, and seeds). Moreover, it is often asserted after liberalization and privatization dismantled many government farm credit programs in the 1990s (Kherallah et al., 2002), that small farmers face severe credit constraints and that this is a cause of low use of external inputs (Kelly et al., 2003, Morris et al., 2007, Poulton et al., 1998, Poulton et al., 2006).

Yet Sheahan and Barrett (2014) find that SSA farmers now purchase more external inputs than in the 1990s, and much more than is generally asserted in the debate. Farmers are thus financing inputs somehow. Is it by credit? If so what kind? Is it by own cash sources from crop sales and labor sales? These issues lead us to the three research questions we address here: (1) how do farmers finance input purchases? (2) Is there a correlation between finance source and farm size and thus “inclusiveness” of the financial arrangement used? (3) Is there a relation with crop type and thus relation to cash crop versus food crops?

To derive hypotheses for these questions, we briefly review the literature concerning the potential finance sources for inputs.

First, government credit was common before the 1990s for both farmers producing cereals as well as export cash crops. The schemes generated fiscal deficits and suffered frequent non-recovery, considered “strategic default” used by farmers as de facto insurance after bad harvests (Poulton et al., 1998). These schemes were generally dismantled in the 1990s and 2000s during Structural Adjustment. We hypothesize that few farmers use government credit now.

Second, government subsidies to farmers to buy fertilizer were common before Structural Adjustment. The subsidy was administered as a reduction of fertilizer price, or as a coupon to farmers (as a direct transfer). Many input subsidy programs were eliminated by Structural Adjustment. However, in several SSA countries they were partially revived in the mid-2000s on the heels of concerns that fertilizer use had dropped since Structural Adjustment. Malawi and Tanzania governments provide many farmers a coupon for fertilizer sufficient for an acre. The Nigerian government had a subsidy scheme during our study period (2010−2012) but our analysis showed only 5% of the farmers bought fertilizer from government sources that disbursed the subsidy.

Third, private-sector banks tend, according to much of the literature, to lend little to farmers (Poulton et al., 1998, Poulton et al., 2010). The reasons given are that banks face high transaction costs in rural areas, farmers tend to lack collateral, and lending is risky because recovery rates are low (Dorward et al., 2009). We hypothesize that few farmers obtain bank credit, but those that do are larger farmers (based on work by Zeller and Sharma (1998) in Cameroon, Ghana, Madagascar, and Malawi).

Fourth, informal credit from friends and family and local moneylenders is often presented as a significant source of funds for farmers to buy inputs and consumption items (Poulton et al., 2006, Zeller and Sharma, 1998). Our hypothesis is thus that informal credit is important to all strata of farmers.

Fifth, finance from “tied output-credit” or “interlinked credit” arrangements (Bardhan, 1980, Poulton et al., 1998) involve an output buyer or input seller advancing the farmer cash for inputs or inputs in kind at the start of the season, and being reimbursed from the farmer’s harvest. The literature presents this in two categories.

The first category is tied output credit from processing or export companies for traditional export cash crops as well as for non-traditional crops like horticulture. The literature is ambiguous as to the occurrence of this. On the one hand, a number of studies especially of particular schemes document this arrangement. On the other hand, some studies note that processing and export companies may not use this arrangement frequently or apply it to all farmers because they fear farmers will “side sell” (to other buyers) or because there is a dearth of effective farmer cooperatives to enforce repayment among their members (Shepherd and Farolfi, 1999, Poulton et al., 1998, Poulton et al., 2010, Chao-Béroff, 2014).

The second category is interlinked credit from grain wholesalers and input dealers. This is commonly posited to be important in Asia (Bardhan, 1980, Conning and Udry, 2007) and in some reports hypothesized to be common in SSA (Pearce, 2003, Zeller and Sharma, 1998).

In both cases farmers enter these “tied” arrangements principally because formal credit markets idiosyncratically fail for them, and thus these are “second best” arrangements (Binswanger and Rosenzweig, 1986). We hypothesize that empirical analysis will show that such arrangements are common in SSA, perhaps with a bias toward traditional cash crops.

A variant on the above is a tied output-labor market arrangement where farm workers advance labor in exchange for payment (typically in kind but can be in cash) at harvest (Bardhan, 1984). While discussion of this was common in the South Asia literature in the 1970s/1980s, to our knowledge it has not been examined empirically in SSA. We hypothesize that it exists in SSA. One justification for this expectation is that labor by one household provided to another is monitored and upheld by local norms/customs and social pressure.

Sixth, household retained earnings such as from rural nonfarm employment and crop sales are in principle candidates for potential liquidity sources for farmers to buy inputs. Indeed, Haggblade et al. (2010) note that rural nonfarm income (RNFI) is a main cash source of rural households in SSA, and Reardon et al., 1994, Davis et al., 2009 hypothesize that RNFI is a key cash source and determinant for input purchases, especially in the face of idiosyncratic failure of credit markets. Yet the empirical literature rarely compares household own-cash sources with credit as potential liquidity sources for farmers to buy inputs. Zeller and Sharma (1998) note that the literature on farm credit is largely independent of the literature on farm household income sources.

However, several studies in SSA provide evidence of the role of RNFI as a finance source for investments of rural households. Aryeetey (1997) provides evidence of the latter for Ghana for rural microenterprises but not for agriculture. Some work has shown the impact of RNFI on external input use by African farmers (e.g., Savadogo et al. (1994) for animal traction in Burkina Faso; Clay et al., 1998, Oseni and Winters, 2009 for fertilizer in Nigeria and Rwanda), and for Asia (e.g., Stampini and Davis (2009) for purchased seeds in Vietnam); some work has shown the effects of off-farm income on farm productivity (such as Rozelle et al., 1999 for China). We thus hypothesize that own cash sources are a significant determinant of input purchases.

The aim of this paper is to examine the above hypotheses and thereby “update the landscape” of knowledge of SSA farm households’ sources of finance for external inputs. To our knowledge, there has been no such survey-based analysis especially over countries using recent and nationally representative data. We analyze recent (2010−2012) LSMS data sets comprising 11,000 farm households in Malawi, Nigeria, Tanzania, and Uganda.

The paper proceeds as follows. Section 2 discusses data and sampling. Section 3 descriptively examines the purchase of “external inputs” and use of credit sources for those purchases, as well as cash income sources. The analysis stratifies by country, farm size, and crop type (using the triad of crop categories in the SSA literature: traditional export crops, non-traditional commercial crops such as horticulture, and staple food grains). Section 3 focuses on Nigeria to econometrically test for the effects of different cash sources on fertilizer demand. The analysis uses panel data estimation techniques to more consistently identify the effect of RNFI on fertilizer demand by accounting for unobserved time invariant household characteristics likely to affect participation in non-farm activities and fertilizer demand. As far as we are aware, there are no other studies that have used nationally representative panel data to explore the effect of non-farm activities on input demand. Most of the older literature (cited above) focused on qualitative analysis, comparison of means and ordinary least square (OLS) estimations that are potentially biased (e.g., Ellis and Freeman, 2004). More recent empirical work such as Oseni and Winters (2009) use cross sectional data while Smale et al. (2016) use panel data but do not use a nationally representative sample (they focus on one maize producing region of Kenya).

## 2 Data

We use data from the Living Standard Measurement Study (LSMS) household panel surveys in four countries. The most recent years of the panels are used for the descriptive analysis in all the countries, and the most recent two years for the econometrics analysis in Nigeria. The sets are as follows: (a) the Malawi Integrated Household Panel Survey (IHPS) of 2012/2013, with 3219 farm households; (b) the second wave of the Nigeria Living Standard Measurement Study – Integrated Survey on Agriculture (LSMS-ISA) Panel for two years, 2010/2011 and 2012/2013, covering 3000 farm households; (c) the Tanzania National Panel Survey 2012/2013, covering 3047 farm households; and (d) the Uganda National Panel Survey 2010/2011 covering 2109 farm households. The surveys differ somewhat in the specific questions they use to elicit information on the variables of interest. We treat the survey datasets as uniformly as possible to ensure that the information is comparable. Where one set or the other lacks some information we note that in the table notes.

In general, the surveys used a two-stage sample design. In the first stage, enumeration areas were selected in each district of the country. Within each enumeration area a listing of households was done for the sample frame. A random sample of households was drawn from that frame. We selected only households doing any farming. In the analyses, we use sampling weights from the datasets to account for the survey design and construct nationally representative statistics. The weight for each household is the inverse of the probability of being selected based on the sample frame structure.

The data used are on farm households’ use of inputs and cash and in-kind arrangements to pay for them. The analysis is done by crop, household, and plot. The data also have characteristics of the farm households such as nonfarm income, crops sales, loans received, and farm size.

## 3 Descriptive analysis of cropping and input purchases

### 3.1 Patterns in cropping

Table 1 shows crop composition by country and farm size strata. Crops are classified into sets: crops traditionally called “food crops” (although they are often also sold for cash), including grains, horticulture products, legumes, and tubers (grown as a staple), and crops traditionally called “cash crops”, including tobacco, cotton, tea/coffee, and edible oil crops.

Several points stand out. First, as expected, grain farming dominates, but is not ubiquitous, as it is practiced by only about three-quarters of the farms in Nigeria, Tanzania, and Uganda, being near 100% only in Malawi. There is little farm size bias in participation in food cropping. Over the countries on average nearly a third of the farms grow horticultural crops, half grow beans/pulses, and a third grow tubers. Food cropping is thus fairly diversified on average.

Second, by contrast, cropping of traditional cash crops is more concentrated in every country. On average, only a fifth of farmers grow traditional cash crops, and that is but a tenth if one excludes Uganda. There is a marked correlation of the share of farms producing any cash crop and farm size. The crop focus differs over countries, with tea/coffee and oil crops standing out in Uganda, cotton and oil crops in Tanzania, oil crops in Nigeria, and tobacco and cotton in Malawi.

### 3.2 Patterns in input purchases

Table 2 shows farmers’ purchases of “external inputs” – variable inputs apart from labor, including inorganic fertilizer, seeds, and pesticides.

First, there is a marked contrast between Nigeria and Malawi, with a high share of farmers buying external inputs (70%), compared to Uganda and Tanzania (16% and 18% respectively). The Malawi-Nigeria results are at odds with the traditional notion that few farmers in SSA use external inputs but consistent with the findings of Sheahan and Barrett (2014).^1^

One might say that the Nigeria and Malawi results are driven by the fertilizer subsidy program. While that might be true in Malawi where about 60% of households receive subsidized fertilizer (Chirwa and Dorward, 2013), this is unlikely in the case of Nigeria. While the Nigeria data show persistently high fertilizer use rates across both survey years rounds, in 2010, when subsidized fertilizer was only channeled through the government, fewer than 5% of the households who purchased fertilizer bought it from government sources (the channel by which the subsidy was delivered).^2^

Second, among farm households buying external inputs, fertilizer and seeds are common purchases. The results are mixed for pesticides. Many farmers buy pesticides in Nigeria, but not in Malawi. Only about a half and a third of the farmers who buy external inputs in Tanzania and Uganda buy fertilizer, yet a larger share buy pesticides; this appears surprising, but is consistent with Sheahan and Barrett (2014) for Uganda.

Table 3 disaggregates input purchases over five strata, very small farmers (with less than 0.5 ha) to larger farmers with more than 5 ha. Several points are salient.

First, across the countries and contrary to conventional perceptions, farmland is concentrated. We find 65–75% of the land but only 20–25% of the farms in the medium and large farm strata (above 2 ha). Small farmers of less than 2 ha have only 25–35% of the land but 75–80% of the farms in Nigeria, Tanzania, and Uganda; in Malawi the farms above two hectares are only 4% of the farms but nearly 40% of the land.

Second, surprisingly, the shares of farmers buying external inputs do not differ much over small (up to 2 ha) versus medium/large (above 2 ha): in Malawi, 71% versus 88%, Nigeria, 78 versus 83%, Tanzania, 15% versus 23%, and for Uganda, 14% versus 24%. But this masks differences in rates, or level of external input use per hectare. Binswanger and Ruttan (1978) note that one should expect smaller farms to use more external inputs as substitute for land. Our data indeed show smaller farmers using more external inputs per hectare than do the medium/large farms: while medium/large farmers crop 70% of total farmland, they constitute only 35% of the external input purchase “pie”. This finding varies little over input types. It also holds true across Malawi, Nigeria, and Uganda. The outlier is Tanzania, where medium/large farms use external inputs almost as intensively as small farms.

### 3.3 Farm input finance by farm size strata and crop categories

Table 4 shows consistent evidence across countries of very low use of any form of credit to buy external inputs. On average, among farm households who bought external inputs, only about 6% used *any* form of credit. As noted in the introduction, there has been a presumption in the literature that to the extent farmers buy external inputs, they do it at least with informal credit or trader credit. But the analysis here shows that conventional wisdom is not supported empirically, and it is not just a lack of formal credit, but a near absence of the use of any credit, formal or informal, tied with input or output traders, in kind or in cash. The converse is that 94% use only their own cash to buy external inputs. This can be from sales of crops and employment earnings (farm wage labor, migration, and RNFI), as discussed in more detail below.

Moreover, among the tiny share of farmers buying external inputs on credit, there is sharp variation over input types. There tends to be 2–3 times more households getting some kind of credit for fertilizer compared to seeds or pesticides.

Table 5a shows the shares of the farm size strata in all credit-based input expenditures. In Malawi, Tanzania, and Uganda, input credit is roughly correlated with farm size – most of the credit-based external input expenditures are concentrated outside the below-one-hectare group. These results do not differ much over input types. Nigeria has the lowest share of farmers purchasing external inputs on credit (3%); it differs somewhat from the other countries in that the great majority of the input credit is taken by the “under 1 ha” group; however, this is still taken by merely a tiny share of the smallest farmers.

Table 5b shows the share of each external input’s expenditure that a given stratum buys with credit. Input credit tends to be much more important for the middle to higher farm size strata, and extremely little for the smaller strata. It is also mainly in fertilizer and very little in pesticides and seeds. In Malawi, Tanzania, and Uganda, input credit is relatively substantial only for fertilizer. It averages 9% of fertilizer input outlay in Malawi but is concentrated in the upper-small and medium farmers (1–5 ha) where it averages a fifth of external input expenditure. In Tanzania, the share of input expenditure done on credit is correlated with land size, with about 10% for smaller farmers and about a quarter and a half for medium and larger farmers. For Uganda, it is only relatively important for the 1–5 ha group, where it reaches 40–50% of fertilizer expenditure. In Nigeria, the share is low for all types of external inputs, with about 3% on average, differing little over strata.

### 3.4 Finance by crop type with an added focus on interlinked credit

Conventional perceptions from the literature, as discussed in the introduction, suggest that farmers growing traditional cash crops would commonly access external inputs on credit, in particular from processors in interlinked credit arrangements; food crop producers also may access such interlinked credit from traders. To test this, we explore the shares (by crop type) of farm plots on which inputs purchased on credit in interlinked relations are shown (Table 6). The findings are surprising.

First, while there is a lot of variation over countries, the average over all traditional cash crops is only 13%, lower than what would be expected from the literature on cash crops that suggests a wide distribution of interlinked credit for cash croppers. This average masks variation over countries. Malawi and Tanzania average 20% (the share, among all plots for this crop category, that receiving inputs purchased in interlinked credit arrangements). Nigeria and Uganda average only 6%.

The difference between the pairs of countries is mainly driven by tobacco in Tanzania and Uganda, where four-fifths of the plots are grown with inputs bought on credit from the processors. Also, that outlier is composed of a tiny group of tobacco farmers in the sample for each country, about 1% of the total sample. The limited and “enclave” nature of tobacco farming and its correlation with farm size in those countries could explain why these are the main cases where the conventional image of contract-farming related credit is manifest. Removing the tobacco outlier (for just Tanzania and Uganda) puts the overall credit share for cash crops about 6% – very close to that for food crops as noted below.

Second, only 6% of all plots of food crops receive inputs purchased in interlinked credit arrangements. This is the first time in the literature this has been tested and demonstrated, and we consider this a key contribution of this paper.

To triangulate the above results on output/input credit arrangements, we examined the data in another section of the LSMS survey questionnaire, the management of crop harvests. We used farmers’ responses concerning use of part of their harvests to repay advances for inputs from input or output traders and processors (especially for cash crops) for external inputs, and for labor.

Table 7 shows the share of farmers using part of their harvests for these ends. The main finding is that such “tied credit” is very rare for external inputs (fewer than 2% of the farmers) across all study countries. This corroborates the results from above. For harvest payment for external inputs, the shares are so small that there are no interesting inter-strata differences. When we consider the “reimbursement of credit with the harvest” by type of crop, it is very minor or zero for the other cash crops (except tobacco in Tanzania, discussed above), and all of the food crops (Table 8).

By contrast, and reported for the first time in the SSA literature using cross-country surveys for comparison, we find that labor-output tying is much more common, with as many as 42% of the farmers in Malawi, 26% of Nigerian, and 68% of Tanzanian farmers doing this practice. (The dataset for Uganda did not allow this calculation.)^3^ The patterns over strata differ by country. In Uganda, the share rises with farm size, in Nigeria it slightly declines, and in Malawi it is in an inverted-U shape relation with farm size. Thus one cannot say that this traditional-tying of labor and harvest is more a phenomenon of the smallest farmers holding on to an old practice, as one might expect, given our hypothesis that larger farms are more apt to use monetized labor relations only. Moreover, Table 8 shows that use of harvest repayment for labor is very minor for cash crops (except for oil crops in Uganda where it is a quarter of farmers using it), but is significant in food crops across the countries, such as about a third in horticulture and a quarter in grains. There is only a single situation (crop plus country) where this arrangement (using harvest to pay for inputs) is important for external inputs, and that is for tobacco in Tanzania. This corroborates the results from above.

We conjecture that this high prevalence of the use of harvest to reimburse for external inputs received on credit to produce tobacco in Tanzania is related to a widespread use of contract farming arrangement over tobacco production in Tanzania. If our conjecture is true, we should expect to see more contract farming (outgrower) arrangements over tobacco compared to cotton, tea/coffee, and oil crops. But in the Tanzania data set,^4^ we found that only 1.8% of farmers are involved in outgrower schemes. In this tiny set, tobacco farmers dominate (as 78% of the plots in outgrower schemes are under tobacco, followed by cotton with 19%.

Overall our results indicate that there is much less tied credit arrangement to finance external input than expected from the conjectures in the literature. Even though those arrangements appear to be more formal (from contract farming arrangements) and more likely for cash crops, we still see far less than expected (except for tobacco).

### 3.5 Households’ use of loans not specifically linked to input transactions

We use the term “loans” for credit unconnected directly and specifically to transactions of outputs or inputs. Loans can come from formal sources (banks), semi-formal sources (micro-finance), and informal sources (friends, relatives, cooperatives, etc.). The survey data show that households take loans, but rarely for agriculture. In Nigeria 38% of the farmers took loans (but there is no information in the survey on the purpose of the loan). In Malawi, 23% of the households took a loan, but only 5% of them did so for farming. In Tanzania, only 11% took loans, of which 2% for farming. This is striking because one would expect credit-constrained farmers to use these loans to finance farm input purchases. For Uganda the survey did not report loans.

Instead, the data show that the loans were taken mainly for nonfarm business startups and non-farm enterprise inputs (40% in Malawi, 24% in Tanzania) and for food consumption (31% in Malawi and Tanzania).^5^ As our regressions show below, a key factor that determines fertilizer purchase is engaging in nonfarm enterprises. Thus it appears that farmers prefer to use loans to finance the set up/expansion of their nonfarm enterprises but use the generated cash from these nonfarm enterprises to finance external input purchases for their farms.

## 4 Determinants of fertilizer purchases in Nigeria

This section infers how farmers finance their input purchase by estimating the determinants of fertilizer purchases by Nigerian farmers. Our analysis emphasizes the roles of the main cash sources of farm households, including RNFI (from both wage and self-employment), crop sales, and loans, in rapidly descending order of importance. We also control for agricultural productivity risks (captured by zone rainfall variability), as well as regional differences (north versus south) in decisions on fertilizer purchase. We focus on the Nigeria case to abstract from possible issues of the fertilizer subsidy directly driving fertilizer purchase, which could be an issue if we were to do the analysis on Malawi and Tanzania as noted above.^6^ We use the two available waves (for 2010 and 2012) of The Nigeria Living Standard Measurement Study-Integrated Survey on Agriculture (LSMS-ISA); the panel version of the nationally representative dataset used in previous sections.

### 4.1 Conceptual and empirical framework

The fertilizer purchase decision follows a standard input demand function derived from a constrained household utility maximization problem (Sadoulet and de Janvry, 1995). Fertilizer demand can be expressed as a function of output and input prices, risk proxies, complementary and substitute farm capital, and relevant shifter variables such as crop type. We consider the decision to purchase fertilizer and then the intensity of use.

In each case

$$Y_{\mathrm{it}}=f(X_{\mathrm{it}}\text{,}u_{\mathrm{it}})$$

where $Y_{\mathrm{it}}$ refers to the binary input use variable or the quantity of fertilizer purchased (in kg), while $X_{\mathrm{it}}$ refers to a vector of controls that explain fertilizer demand. $u_{\mathrm{it}}=\varepsilon_{\mathrm{ijit}}+c_{i}$ is a composite error term comprising time invariant unobservable heterogeneity ($c_{i}$) and time varying unobserved characteristics $\varepsilon_{\mathrm{it}}$ of our input demand function. We model the farmer’s fertilizer purchase decisions using the standard unobserved effects binary dependent variable model (Green, 2000, Wooldridge, 2010). The intensity of fertilizer use is modeled using the unobserved effects Tobit model to account for the corner solution nature of the dependent variable (Wooldridge, 2010). In both models, $c_{i}$ represents the unobserved effect parameter, modeled using the Mundlak (1978) special case of the approach of Chamberlain (1982) called correlated random effects (CRE):

$$c_{i}=\psi +\overset{‾}{X_{i}}\xi +a_{i}\text{,}$$

$$a_{i}|X_{i}∼\mathrm{Normal}(0\text{,}\sigma_{a}^{2})$$

where $\overset{‾}{X_{i}}$ represents time averages of the explanatory variables. The CRE model is preferred over alternative methods such as the fixed effects (FE) and random effects (RE) models in the case of non-linear models (Wooldridge, 2010). However, for comparison, we estimate the linear model with household FE given its suggested conceptual robustness over nonlinear models such as the Probit and Tobit (Angrist and Pischke, 2008).

Consistent with the CRE model, the determinants of the fertilizer purchase decision and the level of use are estimated using pooled Probit and pooled Tobit regressions, respectively. Each regression equation includes a set of explanatory variables as well as the time averages of the explanatory variables. A Wald test of joint significance of the time average variables is performed to test whether a traditional random effects model would be appropriate. A dummy variable for the time period is included to account for time-specific factors that affect fertilizer demand.

Although the use of the FE and CRE models address potential biases due to time invariant unobserved heterogeneity, conditional strict exogeneity implies there is no endogeneity after controlling for the time-invariant unobservables. If this assumption fails our estimates might be biased. To minimize any remaining bias from time-varying unobservables, we include various observable characteristics to proxy for a number of unobservables. Conditional on the covariates used, the likely major source of endogeneity should be time invariant and thus addressed by the CRE approach (corroborated with FE results). However, since it is not possible to completely rule out endogeneity due to time-varying unobservables, these results are interpreted as correlates rather than causal effects.

As an alternative specification, we also consider the likelihood that the decision to work in RNFI, sell crops, or take a loan might be jointly made with the decision to use fertilizer. A farmer may decide to engage in non-farm activities (or take a loan or sell some of his crops) to get cash to purchase inputs including fertilizer. Furthermore, the joint decision process could be due to unobserved characteristics that determine both non-farm participation (taking a loan and selling crops) and input use such as labor availability and networks. Consequently we estimate a seemingly unrelated multivariate Probit regression. Given the non-linearity of our outcome variables and the recursive structure of the model, we do not face the classical identification issue common in linear SUR (Wilde, 2000, Smale et al., 2016). This system approach offers an efficiency gain by taking into account correlations among the residuals of the equations in a system of equations capturing the binary decisions to purchase fertilizer, participate in non-farm self-employment, participate in non-farm wage employment, take a loan, and sell crops).

As in the single equation estimations, we control for specific time-invariant unobservable heterogeneity and include time-varying covariates.

The explanatory variables used in the models and their levels are reported in Table 9. The variable sets and key descriptive points are as follows.

First, three (potential) sources of input finance are included in the model: (1) a dummy variable for RNFI, including self-employment and wage employment; (2) crop sales per hectare of land; and (3) a dummy variable for any member of the household having taken a loan the year before the survey period. Table 9 shows that around 60% of households have at least one member in RNFI self-employment and around 20% with a member with wage employment. The RNFI patterns are similar in the North and South. Table 10 shows that together they are about three-quarters of rural household cash income in 2012. Crop sales in the South were more than double those in the North. In both regions they average about a quarter of cash incomes. Note from Table 10 that livestock sales and remittances are tiny compared with these other sources. Also note that the cash levels of the credit transactions for external inputs are very low compared to cash incomes.

Second, we included several socio-economic variables (gender, age, and education of the household head, as well as the dependency ratio and distance from the market) to proxy for systematic differences in resource access, transaction costs, productive structure, and the number of years of experience in farming (Feder et al., 1985).

Third, we included household-level asset variables, in particular, farm size and agricultural quasi-fixed assets (tractor, plow, irrigation pump, and so on). The latter were captured using an asset index computed using the principal component analysis approach (Filmer and Pritchett, 2001). Note from Table 9 that the farms in the North are roughly double the size of those in the South.

Fourth, we included shares of crop types in the cropped area of the farm. In general, there is much more grain cropping and much less tuber and horticulture cropping in the North compared with the South. This is roughly correlated with rainfall levels.

To account for zone and region effects, we include the following sets of variables.

First, we have dummy variables representing the six main zones (Northeast, Northwest, Southeast, Southcentral, and Southwest), reflecting different infrastructural and growing conditions. Also at a broad level, we have a dummy for urban versus rural areas (as there is farming by households classed in urban areas). In addition to the overall (country level) analysis, we estimate regional-level parameters for the Northern and Southern regions (the sub-regions as noted above). As mentioned by Oseni and Winters (2009), there are important cultural and socio-economic differences between the two regions which can affect the way farmers respond to changes in determinants of inputs use. Table 9 shows that compared to the South, the north of Nigeria is more rural and traditional, with larger household sizes, greater poverty, and less education.

Second, we have several variables at a more disaggregate level, the LGA (the “local government area”). These include the price of fertilizer, and agricultural productivity risk. The latter is captured by the coefficient of variation of rainfall in the LGA, hypothesized to reduce the demand for fertilizer, especially in the absence of ex-post risk mitigation opportunities and lack of credit and insurance mechanisms (Dercon and Christiaensen, 2011).

### 4.2 Regression results

Table 11a, Table 11b present the average partial effects of the determinants of fertilizer purchase overall in Nigeria and by region from the pooled Probit and pooled Tobit estimates. The CRE and FE results are generally consistent and in line with the literature on fertilizer demand. However, they reveal substantial differences between northern and southern Nigeria. Most relevant determinants of fertilizer purchases show higher significance in the North compared to the South. This possibly reflects the North using more fertilizer and therefore is more responsive to various determinants than the South.

We find that participation in non-farm self-employment has positive and significant effects on fertilizer purchases. The estimated APE (Average Partial Effects) indicates that participation in it raises the likelihood of purchasing fertilizer by about 7%. This result is consistent across both the South (10% increase) and the North (5%). These findings coincide generally with the descriptive findings above, and corroborate earlier findings of nonfarm income on input purchase, such as Adesina (1996) for Ivory Coast and Oseni and Winters (2009) for Nigeria. However, contrary to Oseni and Winters (2009), we find that wage employment did not appear as a significant determinant of fertilizer purchase and even has a negative coefficient, perhaps due to wage employment drawing members away from the farm area and thus competing with farming (as Smale et al., 2016 hypothesizes)^7^. Moreover, neither RNFI variable is a significant determinant of the amounts of fertilizer purchased, according to the Tobit results. The balance between farm and non-farm competition for resources on one hand, and the relaxation of cash constraints to allow financing of agriculture inputs on the other hand, determine the observed effects of non-farm employment. In our case, they seem to cancel each other out, especially when we look at the effect on fertilizer amounts.

While lagged access to loans positively affects fertilizer purchase, the effect is significant only in the North. A closer investigation of the types of loans taken by farmers shows that loans from friends and relatives (rather than loan from formal and semi-formal institutions) drive most of these results (with the regressions using different kinds of loans as explanatory variables not shown in the tables). This could illustrate the fact that loans, and in particular loans from formal and semi-formal institutions, are limited for agricultural investment. Given the risks related to agricultural activities, formal and semi-formal credit suppliers are reluctant to provide loans for agricultural purposes, as they fear higher risk of default. Although we could not test specifically this hypothesis in Nigeria due to data limitations, as we noted above, the data for Malawi and Tanzania show that consumption and investment in business start-ups are by far the primary purposes of the loans taken by households. Besides, the fact that the effect of loan is significant only in the North could be explained by the dominant sources of loans in each region. Friends and relatives seem to be a dominant source of loans taken by households in the North compared with the South. Our analysis of the loan data in Nigeria from the LSMS (not shown in a table) provides some evidence for this. There are 22–26% of households reporting loans from friends and relatives in the South, compared to 30–33% in the North.

The coefficient of variation of rainfall has, as expected, a strongly negative effect on fertilizer purchase, but this is only significant in the North. This result is important as investments in modern input use though generally profitable, are costly and can yield very low (or even negative) returns in case of negative weather shocks.

Other factors that significantly affect fertilizer purchase are as expected such as education of the household head with a positive and significant effect in both the North and the South. The farm size effect is significant and positive only in the North, while it is negative but not significant in the South. Crop sales affect positively, but not significantly, the fertilizer purchase decision.

The results of the seemingly unrelated regressions (available upon request from authors, given space limitation) are also consistent with the single equation results. Both the household and geographical factors that affect demand and more importantly the positive effect of non-farm self-employment and loans on fertilizer use are maintained. However, the unexplained portions of the fertilizer purchase equation and the other sources of cash (including self-employment, crop sales and taking a loan) were not correlated for the most part suggesting that these decisions are not necessarily made jointly and thus appropriately modeled using the single equation CRE and FE.

## 5 Conclusions

Many believe that SSA farmers’ increasing their purchase of external inputs such as fertilizer, seed, and pesticides can bring a welcome increase in yields. It has also been observed (Sheahan and Barrett, 2014), and echoed in our paper, that the purchase of these external inputs is definitely no longer absent in SSA and is even very prevalent in some countries, contrary to the common perception. There had not been a systematic exploration of how farmers are paying for these inputs – in particular, what were the relative roles of two sources of cash to pay for inputs (inter alia) – credit (informal and formal) and own cash income. This paper systematically delved into nationally representative datasets for four countries in SSA with widely varying characteristics (Malawi, Nigeria, Tanzania, and Uganda) and examined the roles of these sources.

While the literature emphasized that with the reduction or elimination of parastatal agrarian banks formal bank credit is seldom or never available to Sub-Saharan African farmers for inputs, there was explicitly or implicitly in the literature the working hypothesis that farmers used traditional tied credit with output and input traders, and other sources of informal credit to finance their purchase of external inputs for non-contract farming situations. For cash contract-farming situations and cash cropping in general, the working hypothesis in much of the literature is that processors front inputs or cash for inputs to farmers.

By and large, our paper contradicted these “common wisdoms” concerning the use and role of credit in input purchase. First, we found that very few farmers use any form of credit, formal or informal to finance external input purchase. Second, we found that “tied” credit-output relations are very rare and very minor in external inputs, but especially among smaller farmers in poorer places. What is still significant is tied labor-output markets where local workers advance labor and are paid at the harvest, largely ignored in the literature. Third, we found that generally “traditional cash crop farmers” rarely receive credit from processors, except in a few enclaves like larger tobacco farmers in Tanzania.

Furthermore, we found econometrically that nonfarm self-employment (but not wage employment) plays a significant and positive role in inputs purchase decision, especially given the limited availability of credit for agricultural purposes. It appears that farmers use loans to start nonfarm enterprises (and finance consumption) and plow back the cash partly into their farm input needs; an important observation worthy of further exploration.

These findings do not reflect on or test whether farmers face credit constraints; the fact that farmers use very little credit, informal or formal, for farm inputs, does not inform researchers or policymakers whether the farmers have too little access to credit. What we can say from the data is that nonfarm employment is providing a major source of cash that currently far eclipses use of credit for inputs purchases. When farmers take loans, they mainly use the funds to start nonfarm enterprises or finance consumption. They then often use nonfarm income cash to buy farm inputs. That appears to imply that farmers see that employment as a crucial cash source to meet their farm needs. Further rigorous analysis is necessary to confirm this but it implies that rural development policies and programs that spur broad development of the rural nonfarm sector, in manufacture and services, could benefit farm input purchase and thus productivity and food security, and certainly be an important complement to credit policies and programs.

## Figures and Tables

**Table 1:** Share of households producing key cash and food crops across farm size strata.

Crop types	Farm size strata (ha)	Share of farms with crop (%)			
		Malawi	Nigeria	Tanzania	Uganda
*Cash crops*					
	0–0.49	4	10	4	26
	0.5–0.99	18	10	8	34
	1–1.99	39	11	11	39
	2–4.99	49	20	14	46
	5+	28	14	18	54
	All	17	11	11	37

Food crops					
*Grains*					
	0–0.49	98	69	61	70
	0.5–0.99	99	87	74	83
	1–1.99	99	86	79	86
	2–4.99	99	84	83	81
	5+	100	88	85	81
	All	99	77	76	80

*Horticulture*					
	0–0.49	29	33	22	55
	0.5–0.99	31	21	13	50
	1–1.99	37	22	12	48
	2–4.99	32	23	9	46
	5+	43	17	7	63
	All	31	28	13	51

*Legumes*					
	0–0.49	62	29	12	76
	0.5–0.99	76	56	10	75
	1–1.99	79	60	12	77
	2–4.99	77	53	16	82
	5+	93	54	16	82
	All	71	42	13	78

*Tubers*					
	0–0.49	8	61	16	74
	0.5–0.99	9	30	19	79
	1–1.99	14	34	19	74
	2–4.99	16	39	18	76
	5+	0	49	20	71
	All	10	48	18	75

*All food crops*					
	0–0.49	100	98	95	100
	0.5–0.99	100	98	97	99
	1–1.99	100	99	96	100
	2–4.99	100	98	95	100
	5+	100	99	97	99
	All	100	98	96	100

**Table 2:** Share of farm households who purchase external inputs.

Countries	Farm Households buying external inputs (%)	Farm Households (%) by type of external inputs purchased		
		Fertilizers	Pesticides	Seeds
Malawi	70	49	4	51
Nigeria	71	42	38	29
Tanzania	18	8	13	NA
Uganda	16	5	14	NA

*Note:* NA means information is unavailable in the dataset.

**Table 3:** Purchase of external inputs by farm size strata.

	Farm strata (ha)	Farms in stratum (%)	Farmland in stratum (%)	Farms buying external inputs (%)	Fertilizer bought by stratum (%)	Pesticides bought by stratum (%)	Seed bought by stratum (%)	Total inputs bought by stratum (%)
*Malawi*								
	0–0.49	45	13	65	30	12	28	30
	0.5–0.99	33	24	69	21	11	34	22
	1–1.99	18	24	79	29	40	23	29
	2–4.99	4	11	91	19	30	13	19
	5+	0	27	84	1	7	2	1
	Overall	100	100		100	100	100	100

*Nigeria*								
	0–0.49	53	8	62	30	19	55	30
	0.5–0.99	20	12	78	25	20	17	23
	1–1.99	15	16	83	23	24	13	22
	2–4.99	9	22	82	16	21	8	16
	5+	3	43	85	5	16	7	8
	Overall	100	100		100	100	100	100

*Tanzania*								
	0–0.49	20	2	13	5	5	NA	5
	0.5–0.99	19	5	14	9	7	NA	9
	1–1.99	24	14	17	20	13	NA	19
	2–4.99	26	32	22	41	46	NA	42
	5+	11	47	24	25	29	NA	26
	Overall	100	100		100	100	NA	100

*Uganda*								
	0–0.49	26	4	6	6	5	NA	5
	0.5–0.99	24	10	16	9	10	NA	10
	1–1.99	26	20	20	35	48	NA	44
	2–4.99	19	30	20	34	25	NA	28
	5+	6	37	28	16	12	NA	14

*Note:* NA, information unavailable in the dataset.

External inputs include fertilizer, seeds and pesticides.

**Table 4:** Share of households purchasing external inputs that finance the purchase on credit.

	Of those who bought external inputs, share buying on credit (%)	Of those who bought the noted input, share who bought on credit by input type		
		Fertilizers	Pesticides	Seeds
Malawi	5	5	7	3
Nigeria	3	2	NA	3
Tanzania	11	14	7	3
Uganda	6	14	4	NA

*Note:* NA implies information unavailable in the dataset.

Column 2 is the share among households who purchased at least one external input.

**Table 5a:** Credit-based expenditure on external inputs, by shares of strata.

Countries	Farm size strata	Buying on credit (%)	In all credit-based fertilizer outlay	In all credit-based pesticide outlay	In all credit-based seed outlay	In all credit-based input outlay
*Malawi*						
	0–0.49	3	4	11	13	4
	0.5–0.99	3	4	15	16	4
	1–1.99	10	61	38	44	60
	2–4.99	10	32	36	27	32
	5+	14	0	0	0	0
	Overall		100	100	100	100

*Nigeria*						
	0–0.49	3	49	NA	13	45
	0.5–0.99	5	22	NA	22	22
	1–1.99	4	11	NA	62	16
	2–4.99	1	2	NA	0	2
	5+	6	16	NA	3	14
	Overall		100	NA	100	100

*Tanzania*						
	0–0.49	2	0	0	NA	0
	0.5–0.99	6	4	3	NA	4
	1–1.99	8	10	15	NA	10
	2–4.99	20	36	69	NA	38
	5+	24	50	12	NA	48
	Overall		100	100	NA	100

*Uganda*						
	0–0.49	0	0	0	NA	0
	0.5–0.99	2	3	17	NA	5
	1–1.99	11	57	54	NA	56
	2–4.99	11	40	28	NA	39
	5+	0	0	0	NA	0
	Overall		100	100	NA	100

*Note:* NA implies information unavailable from dataset.

Column 3 pertains to farm households buying at least one external input.

**Table 5b:** Share of credit-based outlay in overall outlay per external input.

Countries	Farm size strata	Credit-based outlay in total fertilizer outlay (%)	Credit-based outlay in total pesticide outlay (%)	Credit-based outlay in total seed outlay (%)	Credit-based outlay in total ext. input outlay (%)
*Malawi*					
	0–0.49	1	3	2	1
	0.5–0.99	2	5	2	2
	1–1.99	22	4	8	21
	2–4.99	18	4	8	17
	5+	0	0	0	0

*Nigeria*					
	0–0.49	6	NA	1	4
	0.5–0.99	3	NA	3	3
	1–1.99	2	NA	12	2
	2–4.99	1	NA	0	0
	5+	11	NA	1	5

*Tanzania*					
	0–0.49	2	0	NA	2
	0.5–0.99	12	4	NA	11
	1–1.99	15	10	NA	14
	2–4.99	26	12	NA	23
	5+	58	3	NA	48

*Uganda*					
	0–0.49	0	0	NA	0
	0.5–0.99	12	3	NA	6
	1–1.99	53	2	NA	17
	2–4.99	40	2	NA	19
	5+	0	0	NA	0

*Note:* NA implies information unavailable in the dataset.

Column 3 is among households who purchased at least one external input.

**Table 6:** Share of plots on which external inputs purchased with interlinked credit, by crop type.

	Malawi	Nigeria	Tanzania	Uganda
*Cash crops*				
Tobacco	16	NA	87	81
Cotton	11	8	11	0
Tea/coffee	NA	NA	22	1
Oil crops	6	3	4	11
All cash crops	14	4	26	8

*Food crops*				
Grains	5	3	11	7
Horticulture	4	3	0	4
Legumes	5	2	11	6
Tubers	7	3	4	5
All food crops	5	3	10	6

NA implies information unavailable in dataset.

**Table 7:** Shares of farmers using harvest to reimburse input credit.

Countries	Farm size strata	Share of farmers using their harvest to repay labor received on credit (%)	Share of farmers using their harvest to repay external inputs received on credit (%)
*Malawi*			
	0–0.49	37	1
	0.5–0.99	45	3
	1–1.99	50	2
	2–4.99	47	1
	5+	24	0
	All	42	1.8

*Nigeria*			
	0–0.49	26	1
	0.5–0.99	29	1
	1–1.99	26	3
	2–4.99	21	2
	5+	22	3
	All	26	1.4

*Tanzania*			
	0–0.49	NA	0
	0.5–0.99	NA	1
	1–1.99	NA	1
	2–4.99	NA	4
	5+	NA	5
	All	NA	1.9

*Uganda*			
	0–0.49	54	NA
	0.5–0.99	63	NA
	1–1.99	74	NA
	2–4.99	78	NA
	5+	81	NA
	All	68	NA

*Notes:* NA implies that information is unavailable in the dataset used.

**Table 8:** Financing inputs on credit with harvest across key cash and food crops.

Crops types	Share of plots where harvest is used to repay (advanced) labor (%)				Share of plots where harvest is used to repay external inputs (%)			
	Nigeria	Malawi	Uganda	Tanzania	Nigeria	Malawi	Uganda	Tanzania
*Cash crops*								
Tobacco	0	2	0	NA	0	2	NA	79
Cotton	10	0	0	NA	0	1	NA	6
Tea/coffee	NA	NA	1	NA	NA	NA	NA	3
Oil crops	8	0	25	NA	0	0	NA	0

*Food crops*								
Grains	17	22	27	NA	1	1	NA	1
Horticulture	18	32	36	NA	1	0	NA	0
Legumes	9	21	25	NA	1	1	NA	0
Tubers	5	29	30	NA	1	1	NA	0

*Notes:* NA implies that information is unavailable in the dataset used.

**Table 9:** Summary statistics of variables used in the regression analysis, Nigeria, South, North.

Variables	Nigeria		South		North	
	2010	2012	2010	2012	2010	2012
Household head is Male (0/1)	88	87	76	74	96	96
Age of the household head (years)	51	52	56	57	47	49
Household dependency ratio	1.1	1	0.9	0.8	1.2	1.1
Household head has formal education (0/1)	60	60	71	70	52	53
Household resides in an urban area (0/1)	13	12	18	17	10	8
Land holding size (hectares)	0.9	0.8	0.5	0.4	1.2	1
Agricultural assets index	0.3	0.2	0.4	0.1	0.2	0.3

A household member is engaged in Non-Farm self-employment (0/1)	56	60	51	57	60	62
A household member is engaged in off Farm wage employment (0/1)	23	18	24	23	23	15
Household received any loan (0/1)	39	40	36	42	42	39
Household received loan from formal source (0/1)	3	5	3	8	3	3
Household received loan from informal source (0/1)	18	19	18	24	18	16
Household received loan from friends or relatives (0/1)	28	29	22	26	33	30
Value of sales per ha of land cultivated (in 000 Naira)	43	43	65	69	30	30

Use fertilizer (0/1)	45	45	25	21	59	61
Purchase Fertilizer (0/1)	41	42	23	20	55	56
Fertilizer price (in Naira per kg)	85	103	93	106	80	100
Distance to Nearest Market (km)	71	70	66	66	75	73
Coefficient of variation of rainfall	94	95	68	68	111	112

Share of land cultivated allocated to grains crops	43	44	15	16	59	59
Share of land cultivated allocated to legumes crops	16	17	1	1	25	25
Share of land cultivated allocated to tubers crops	28	25	58	53	10	10
Share of land cultivated allocated to oil crops	3	3	5	6	2	2
Share of land cultivated allocated to horticulture crops	7	8	15	18	3	3
Share of land cultivated allocated to cotton	0	0	0	0	0	0
Share of land cultivated allocated to tobacco	0	0	0	0	0	0
Share of land cultivated allocated to tea/coffee	0	0	0	0	0	0
Share of land cultivated allocated to other crops	3	3	7	7	1	0

Geographic zones						
North central	17	17	0	0	29	27
North east	20	20	0	0	34	34
North west	22	24	0	0	37	40
South east	20	19	49	49	0	0
South south	13	13	31	32	0	0
South west	9	7	21	19	0	0

*Note:* Means of binary variables are expressed in percentage.

**Table 10:** Sources of cash income in Nigeria, North, and South, 2012.

Income sources	Household cash sources (000 naira)			Share of cash from each source (%)		
	Nigeria	South	North	Nigeria	South	North
*Cash income*						
Profit from household enterprises	119.9	110.2	127	46.2	38.0	53.5
Wage income	77.7	105.4	57.3	29.9	36.3	24.1
Crop sales (gross)	60	71.4	51.7	23.1	24.6	21.8
Livestock net sales	1	1.1	0.9	0.4	0.4	0.4
Remittances	1.1	2	0.4	0.4	0.7	0.2

Total cash	259.6	290.1	237.3	100.0	100.0	100.0

Inputs credit transactions	0.4	0.1	0.6	0.2	0.03	0.3
Inputs non credit transactions	10.8	4.2	15.6	4.2	1.4	6.6
Total input purchase	11.1	4.2	16.2	4.3	1.4	6.8
Hired labor value for harvest only	12.9	7.5	16.9	5.0	2.6	7.1
Imputed value of own crop output	140.5	88.7	178.6	54.1	30.6	75.3

*Note:* The numbers in the left panel are zero-in averages. The shares on the right are based on ratio of number on the left to the total cash value. Inputs include fertilizer, seeds, and pesticides. For each value in the table, instead of deleting outliers we winsorized them i.e. replace top 10% values by the highest value within 90% of the distributions, thus creating a pile up at the top without changing the distribution (Cox, 2006).

For imputation of value of own crop output method, we estimate unit prices of crops for crops that were sold, and then we use the median price in the local governments and multiply by harvest quantities to get the value of crop sales.

The harvest labor for planting activities is missing in the 2010 dataset, and therefore we focus on the harvest labor only in both years.

The values reported for the cash sources are nominal values for each year.

**Table 11a:** Estimation results of determinants of fertilizer purchase (0/1) decision.

Variables	Nigeria		South		North	
	CRE Probit	Linear FE	CRE Probit	Linear FE	CRE Probit	Linear FE
Household head is Male (0/1)	0.050^∗^	0.115	0.026	−0.010	0.131^∗∗∗^	0.637^∗∗^
	[0.058]	[0.177]	[0.355]	[0.861]	[0.004]	[0.049]
Age of the household head (years)	0.000	−0.001	0.000	−0.001	0.000	−0.001
	[0.740]	[0.725]	[0.654]	[0.784]	[0.876]	[0.716]
Household dependency ratio	−0.006	−0.012	0.030	0.028	−0.030^∗^	−0.034^∗^
	[0.669]	[0.446]	[0.169]	[0.225]	[0.052]	[0.081]
Household head has formal education (0/1)	0.081^∗∗∗^	0.051^∗∗^	0.056^∗∗^	0.040	0.094^∗∗∗^	0.057^∗∗^
	[0.000]	[0.041]	[0.046]	[0.521]	[0.000]	[0.035]
Land holding size (hectares)	0.017	0.026^∗^	−0.033	−0.013	0.031^∗∗^	0.037^∗∗^
	[0.220]	[0.057]	[0.260]	[0.603]	[0.036]	[0.020]
Agricultural asset index	0.002	0.002	0.003	0.001	0.002	0.002
	[0.267]	[0.323]	[0.403]	[0.576]	[0.349]	[0.315]
Total Livestock Units index	0.652^∗∗^	0.413	−0.649	0.355	0.651^∗^	0.349
	[0.049]	[0.158]	[0.656]	[0.536]	[0.080]	[0.257]
LOG of crop sales in naira per ha of harvested land	0.001+	0.001^∗^	0.000	0.000	0.001^∗^	0.001^∗∗^
	[0.114]	[0.071]	[0.623]	[0.597]	[0.083]	[0.049]
A household member is engaged in Non-Farm Self-employment (1/0)	0.070^∗∗^	0.078^∗∗∗^	0.106^∗∗^	0.135^∗∗∗^	0.052+	0.062^∗^
	[0.012]	[0.009]	[0.020]	[0.009]	[0.149]	[0.078]
A household member is engaged in wage employment (1/0)	−0.022	−0.019	−0.025	−0.022	−0.006	−0.004
	[0.418]	[0.545]	[0.505]	[0.595]	[0.876]	[0.918]
A household member took a loan (0/1)	0.056^∗∗∗^	0.060^∗∗∗^	0.045	0.047	0.069^∗∗∗^	0.076^∗∗∗^
	[0.009]	[0.001]	[0.203]	[0.163]	[0.009]	[0.001]
Coefficient of variation of rainfall	−0.003^∗∗∗^		−0.003		−0.004^∗∗∗^	
	[0.001]		[0.623]		[0.000]	
LOG of fertilizer price in Naira per kg	−0.017	−0.019	−0.015	−0.003	−0.017	−0.033
	[0.616]	[0.481]	[0.729]	[0.920]	[0.737]	[0.438]
Share of total land cultivated allocated to grains crops	0.002	0.003^∗^	0.000	−0.000	0.004^∗∗^	0.003^∗∗^
	[0.171]	[0.061]	[0.813]	[0.711]	[0.047]	[0.047]
Share of total land cultivated allocated to legumes crops	0.001	0.002	0.001	0.000	0.003+	0.002
	[0.492]	[0.173]	[0.478]	[0.829]	[0.138]	[0.199]
Share of total land cultivated allocated to tubers crops	0.001	0.002	−0.000	−0.001	0.004^∗^	0.002
	[0.560]	[0.207]	[0.766]	[0.217]	[0.072]	[0.179]
Share of total land cultivated allocated to oil crops	0.001	0.002	0.000	−0.000	0.002	0.000
	[0.558]	[0.267]	[0.809]	[0.979]	[0.560]	[0.981]
Share of total land cultivated allocated to horticulture crops	0.002^∗^	0.003^∗^	0.001	0.001	0.004^∗^	0.003+
	[0.092]	[0.052]	[0.360]	[0.380]	[0.091]	[0.123]
Share of total land cultivated allocated to cotton	−0.002				0.001	
	[0.387]				[0.774]	
Urban dummy variable (0/1)	0.089^∗∗∗^	−0.096	0.092^∗∗∗^	−0.118	0.050	−0.143
	[0.005]	[0.701]	[0.008]	[0.189]	[0.252]	[0.607]
Household Distance in (KMs) to Nearest Market	−0.002^∗∗∗^	−0.003	−0.000	−0.000	−0.003^∗∗∗^	−0.006
	[0.000]	[0.771]	[0.454]	[0.990]	[0.000]	[0.259]
Year 2010 (0/1)	0.017	0.017	0.029	0.054^∗∗∗^	0.005	−0.005
	[0.237]	[0.191]	[0.154]	[0.004]	[0.784]	[0.767]

*Zone dummies*						
North east	0.095^∗^				0.071	
	[0.057]				[0.185]	
North west	0.324^∗∗∗^				0.310^∗∗∗^	
	[0.000]				[0.000]	
South east	−0.165^∗∗∗^		0.255^∗∗∗^			
	[0.006]		[0.000]			
South center	−0.229^∗∗∗^		0.169^∗∗^			
	[0.002]		[0.011]			
South west	−0.400^∗∗∗^					
	[0.000]					
Constant		0.339		0.168		0.231
		[0.662]		[0.875]		[0.669]

Number of observations	4843	4843	1670	1670	3173	3173
R-squared		0.027		0.045		0.037
Number of households		2730		995		1735

*Note:*^∗∗∗^, ^∗∗^, ^∗^, and + indicate that the corresponding regression coefficients are statistically significant at the 1%, 5%, 10%, and 15% levels, respectively. Model estimated using partial MLE estimation method. P values based on clustered standard errors between brackets. CRE stands for Correlated Random Effects while FE stands for Fixed Effects.

**Table 11b:** Estimation results of determinants of quantity of fertilizers purchased by farmers in Nigeria.

Variables	Nigeria		South		North	
	CRE Tobit	Linear FE	CRE Tobit	Linear FE	CRE Tobit	Linear FE
Household head is Male (0/1)	65.803^∗∗∗^	139.042^∗∗^	20.022	11.096	109.283^∗∗∗^	346.443^∗∗^
	[0.003]	[0.032]	[0.333]	[0.870]	[0.007]	[0.038]
Age of the household head (years)	0.086	−1.871	0.220	0.828	0.319	−2.984
	[0.802]	[0.297]	[0.698]	[0.619]	[0.462]	[0.200]
Household dependency ratio	0.941	4.056	24.422	10.978	−11.982	−0.741
	[0.935]	[0.847]	[0.184]	[0.727]	[0.414]	[0.978]
Household head has formal education (0/1)	31.943^∗∗^	1.798	43.532^∗∗^	8.492	29.495^∗^	9.200
	[0.014]	[0.953]	[0.032]	[0.910]	[0.079]	[0.782]
Land holding size (hectares)	−49.135^∗∗∗^	−111.516^∗∗∗^	−44.826^∗^	−81.532^∗∗∗^	−59.393^∗∗∗^	−117.974^∗∗∗^
	[0.000]	[0.000]	[0.051]	[0.001]	[0.000]	[0.000]
Agricultural asset index	1.779^∗^	1.372	1.948	0.838	1.721+	0.915
	[0.063]	[0.228]	[0.405]	[0.511]	[0.113]	[0.599]
Total Livestock Units index	794.160^∗∗∗^	890.156^∗∗^	−254.360	174.590	843.723^∗∗∗^	833.816^∗∗^
	[0.001]	[0.012]	[0.832]	[0.812]	[0.002]	[0.022]
LOG of crop sales in naira per ha of harvested land	0.277	0.046	0.048	−0.164	0.531	0.433
	[0.536]	[0.940]	[0.936]	[0.859]	[0.402]	[0.571]
A household member is engaged in Non-Farm Self-employment (1/0)	16.582	−12.894	57.657+	2.836	7.946	−5.306
	[0.484]	[0.750]	[0.144]	[0.965]	[0.798]	[0.917]
A household member is engaged in wage employment (1/0)	85.647	12.824	−12.627	16.299	16.685	31.760
	[0.796]	[0.690]	[0.702]	[0.779]	[0.490]	[0.404]
A household member took a loan (0/1)	15.764	3.137	32.709	44.667	14.671	−4.289
	[0.341]	[0.893]	[0.218]	[0.234]	[0.479]	[0.884]
Coefficient of variation of rainfall	−2.147^∗∗∗^		−4.164		−2.568^∗∗∗^	
	[0.000]		[0.356]		[0.000]	
LOG of fertilizer price in Naira per kg	−31.291	−56.164^∗∗^	−35.938	−60.673^∗∗^	−28.953	−64.421+
	[0.208]	[0.024]	[0.293]	[0.041]	[0.437]	[0.114]
Share of total land cultivated allocated to grains crops	1.088	18.904^∗∗∗^	−0.008	−1.120+	2.802^∗^	18.990^∗∗∗^
	[0.224]	[0.005]	[0.993]	[0.147]	[0.074]	[0.004]
Share of total land cultivated allocated to legumes crops	1.019	19.196^∗∗∗^	0.926	−0.400	2.504+	18.899^∗∗∗^
	[0.301]	[0.005]	[0.488]	[0.685]	[0.111]	[0.005]
Share of total land cultivated allocated to tubers crops	0.809	19.181^∗∗∗^	0.001	−0.121	2.074	18.070^∗∗∗^
	[0.375]	[0.004]	[0.999]	[0.805]	[0.167]	[0.006]
Share of total land cultivated allocated to oil crops	1.286	19.935^∗∗∗^	0.665	1.016	1.557	18.326^∗∗∗^
	[0.228]	[0.003]	[0.526]	[0.250]	[0.413]	[0.005]
Share of total land cultivated allocated to horticulture crops	1.623^∗^	19.315^∗∗∗^	0.956	0.116	2.441	18.318^∗∗∗^
	[0.078]	[0.004]	[0.318]	[0.802]	[0.161]	[0.006]
Share of total land cultivated allocated to cotton	−7.072^∗∗∗^				−7.280^∗∗^	
	[0.009]				[0.041]	
Urban dummy variable (0/1)	48.053^∗∗^	−229.742	57.901^∗∗^	−99.588+	18.113	−255.808
	[0.012]	[0.339]	[0.019]	[0.146]	[0.426]	[0.344]
Household Distance in (KMs) to Nearest Market	−1.074^∗∗∗^	1.853	−0.435	3.524	−1.208^∗∗∗^	2.646
	[0.000]	[0.538]	[0.302]	[0.326]	[0.000]	[0.544]
Year 2010 (0/1)	20.286^∗^	10.804	39.974^∗∗∗^	92.170^∗∗∗^	1.296	−30.360
	[0.070]	[0.559]	[0.009]	[0.000]	[0.938]	[0.265]
Zone dummies	72.740^∗∗∗^				54.593^∗^	
	[0.009]				[0.072]	
North east	179.017^∗∗∗^				172.165^∗∗∗^	
	[0.000]				[0.000]	
North west	−32.435		211.842^∗∗∗^			
	[0.425]		[0.000]			
South east	−155.953^∗∗∗^		105.926^∗∗^			
	[0.003]		[0.021]			
South center	−241.589^∗∗∗^					
	[0.000]					
South west		−1551.668^∗∗^		89.799		−1654.857^∗∗^
		[0.031]		[0.755]		[0.031]

Number of observations	4843	4843	1670	1670	3173	3173
R-squared		0.059		0.050		0.083
Number of households		2730		995		1735

*Note:*^∗∗∗^, ^∗∗^, ^∗^, and + indicate that the corresponding regression coefficients are statistically significant at the 1%, 5%, 10%, and 15% levels, respectively. Model estimated using partial MLE estimation method. P values based on clustered standard errors between brackets.

## Footnotes

1 They covered Ethiopia, Malawi, Niger, Nigeria, Tanzania, and Uganda.

2 There is no explicit question in the Nigeria LSMS for whether a household got a fertilizer subsidy. However, until recently, only the government sold subsidized fertilizer; thus we assume that farmers buying from government sources are the only ones getting a subsidy (based on Takeshima and Nkonya, 2014). While this might be an underestimate in 2012 (since it was possible starting in 2012 for farmers to purchase subsidized fertilizer from dealers in the market with a coupon) this is unlikely since the new program was still very new (launched in 2012). We find the very low numbers (and a tiny share) of farmers purchasing fertilizer from government sources in 2012 to be similar to those in 2010 (when the government was the sole distributor of fertilizer).

3 Of interest (but not reported in Table 7) is that tying land access and output markets was not found to be common. That is, the land tenure section of the surveys showed that sharecropping was extremely limited.

4 There is no information about contract farming in the datasets of the other countries to allow us to compare this pattern across countries.

5 Zeller and Sharma (1998) also found that 50–90% of formal and informal loans in their African study countries went to consumption-related purchases. Poulton et al. (2010) also make this point in a general way.

6 Even in 2012, when it was possible that farmers purchased subsidized fertilizer from agro-dealers in the private market less than 5% of farmers could have done so. According to the Federal Ministry of Agriculture and Rural Development about 1.6 million farmers participated in the government subsidy program in 2012 (FMARD, 2015). According to the LSMS-ISA surveys, there were over 32.5 million households in Nigeria in 2012. Even if we assume the program only allowed 1 participant per household, this would amount to about 5% of farmers.

7 This is supported by our data as non-farm wage employment participants supply statistically significantly lower amount of labor a week, on average to agriculture (11.12 h) than non-participants (18.24) with a p value of 0.000.
